# Ovarian Pregnancy

**Published:** 1863-02

**Authors:** R. B. Treat

**Affiliations:** Janesville, Wis.


ARTICLE V.

### OVARIAN PREGNANCY.

By R. B. TREAT, M.D., of Janesville, Wis.

On the 14th of May, 1860, I was solicited to visit Mrs. H., a highly respected, married lady of this city, about 37 years of age, and the mother of several children. I found her much reduced from uterine hemorrhage, which still continued. She supposed herself two months advanced in utero-gestation, when the flooding came on. She also concluded that the fœtus had been expelled, but not the placenta.

Not being satisfied that either had been expelled, I made a careful examination: found the uterus larger than in the unimpregnated state, but in the natural position, cervix shortened somewhat, and the os uteri slightly dilated, but impervious to the finger, consequently, I prescribed the usual remedies for flooding, with rest, and the recumbent position. The hemorrhage gradually ceased, but severe pain continued in the left iliac region, extending down the inner side of the thigh of the same side. Bowels constipated, abdomen extremely tender and tympanitic, precluding a satisfactory external examination. Slight chills, and fever of a remittent type followed. The conjunctiva was deeply tinged with bile; and the entire surface of the body presented a peculiar sallow hue. Under an alterative and tonic treatment, all these symptoms yielded readily; and in two weeks, she was about the house overseeing her household duties.

On the first day of June, I was called again, as she had had some flowing during the night, while using the vessel. It having ceased, I made no vaginal examination, but left particular word that should it occur again, to save the fluid for my inspection, so that I might determine its character. I saw her every day or two, until the 8th day of June, as the pain and fever returned as before. These symptoms subsided in a few days under previous treatment. I did not see her again until the first of July. Several days previous, she experienced much difficulty in voiding urine, and the bowels were obstinately obstructed, which symptoms still continued. I found that the mammary glands had increased in size, and were quite sensitive. The areola was discolored,—papilla prominent. Morning sickness had been present two or three weeks; and the abdomen had gradually enlarged. An examination, par vaginum, detected the cervix uteri high up under the pubic arch, and materially shortened; and the fundus of the organ, I concluded, lay in the hollow of the sacrum. The case now presented to me every indication of pregnancy with retroversion uteri, consequently, I attempted to replace the uterus to its natural position, but was unsuccessful, as the sequel will show. On the 5th and 9th of July, I renewed the effort to replace the organ, but failed. I was now undecided, whether it was a case of pregnancy with retroversion, or extra uterine pregnancy.

I explained fully to her the danger attending in either case; and as she was able to be up, I advised a nourishing, easily digested diet, rest and quietness of mind and body; fully determined if it should be pregnancy with retroversion to interfere at the proper time. The difficulty of voiding urine continued, but the catheter was not required. The bowels were relieved daily by enemas.

I saw the case again on the 2d of August, she having had a sanguineous discharge from the vagina of nearly one pint at once, which produced syncope. Upon enquiry, I found that she had been at work several days, contrary to advice. She said that she had been in usual health, and thought it could do her no harm. Upon examining the fluid, and from all the attendant symptoms and circumstances, I became satisfied the case was one of pregnancy outside of the uterus. The parts and position of tumor were unchanged, although enlarged. The uterus could also be detached in the right iliac region, into which I introduced a sound, which plainly evidenced the fact that the organ had no connection with the tumor, confirming my opinion, that it was really a case of ovarian pregnancy. From the firmness and extent of the adhesion, that I concluded must exist, and her debilitated condition, I saw no relief, not even from surgery. She remained in about the same condition, when she was again attacked with pain, attended by frequent discharges of a bloody serum. I had frequently requested counsel, but they did not desire it; but now, at my urgent request, two or three physicians were called in, who, after making all proper examinations, pronounced it a case of pregnancy with retroversion uteri, until I introduced the sound and showed them the relative position of the uterus and the tumor.

One of the three only, failed to coincide in the diagnosis; and in an attempt to replace the supposed retroverted organ, (against my urgent request,) the cyst was ruptured, and a large portion of its fluid contents passed into the peritoneal cavity, producing death in a few hours. The case being new to me, and rarely met with by the profession, I solicited and obtained the privilege of making a *post mortem* examination, which I performed ten hours after death.

Appearances:—Decomposition had gone on rapidly, and, of course, the abdomen was largely distended, which, on being opened, was found to contain several pints of fluid, of a sanguineous character, not unlike the discharges previous to death, and evidently a portion of the contents of the cyst. The rupture in the cyst was small, and about the centre of it. The adhesions below, were nearly five inches in diameter, and very firm. The half of the fallopian tube, including the fimbriated extremity, was incorporated in the tumor, and might, possibly, account for the flow from the uterus, before death. The canal in the tube was increased in size. Upon opening the cyst, which seemed to be the membranes of a proper pregnancy, thickened and much firmer; a fœtus of six months with placenta, and nearly a quart of bloody serum and some coagula were discovered. The entire cyst and contents were now removed and examined, presenting all the appearances, in every respect, of a proper uterine development. The fœtus was also far advanced in decomposition, which must have taken place within two weeks, as the symptoms of pregnancy remained prominent up to that time. The uterine walls were much thicker than in the unimpregnated state, and very flabby. The cavity also much larger, but contained no fluid. The uterus had no attachments to the tumor, except by the left fallopian tube. The ovary of the right side natural and healthy. I concluded that cases of this kind are seldom met with in long years of practice, at least, I deem such to be the fact, for it is my first; and I am quite confident none have occurred in this vicinity in the practice of any other physician in the past fifteen years. I concluded, therefore, to write more fully the history, symptoms, and fatal termination of this case. It gave me much anxiety, and required much study and thought in determining its true character.

Some readers may conclude that a correct diagnosis should have been arrived at much easier; but, if any such there be, who shall be so unfortunate as to meet such a case, they will find it no easy matter; and if the above truthful and unreserved report shall aid any one in forming a correct conclusion in a case so infrequent in its occurrence, I shall be content.

